# Insulin resistance in lean and overweight non-diabetic Caucasian adults: Study of its relationship with liver triglyceride content, waist circumference and BMI

**DOI:** 10.1371/journal.pone.0192663

**Published:** 2018-02-09

**Authors:** Jorge Gonzalez-Cantero, Jose Luis Martin-Rodriguez, Alvaro Gonzalez-Cantero, Juan Pedro Arrebola, Jorge Luis Gonzalez-Calvin

**Affiliations:** 1 Department of Radiology, HGU Gregorio Marañón, Madrid, Spain; 2 University of Granada, Granada, Spain; 3 Department of Radiology, University Hospital San Cecilio, Granada, Spain; 4 Department of Dermatology, Complejo Hospitalario de Toledo, Toledo, Castilla-La Mancha, Spain; 5 Complejo Hospitalario Universitario de Granada, Instituto de Investigación Biosanitaria ibs CIBERESP, Granada, Spain; 6 Department of Gastroenterology, University Hospital San Cecilio, Granada, Spain; University College London, UNITED KINGDOM

## Abstract

**Aims:**

Insulin resistance is the pathophysiological precursor of type 2 diabetes mellitus (DM-2), and its relationship with non-alcoholic fatty liver disease (NAFLD) has been widely studied in patients with obesity or metabolic syndrome using not only ultrasound but also liver biopsies or proton magnetic resonance spectroscopy (H^1^-MRS) to assess liver fat content. In contrast, there are no studies on insulin resistance and NAFLD in lean or overweight Caucasian individuals using H^1^-MRS or liver biopsies for the quantification of hepatic triglyceride content. Our objectives were to study the presence of insulin resistance in lean and overweight Caucasian adults and investigate its possible relationship with liver triglyceride content, waist circumference (as proxy of visceral adiposity), BMI, and cardiometabolic risk factors.

**Methods:**

A cross-sectional study was conducted in 113 non-obese, non-diabetic individuals classified as overweight (BMI 25–29.9 kg/m^2^) or lean (BMI 19.5–24.9 kg/m^2^). Hepatic triglyceride content was quantified by 3T H^1^-MRS. NAFLD was defined as hepatic triglyceride content >5.56%. Insulin resistance (HOMA-IR), serum adiponectin, and tumor necrosis factor (TNF) were determined.

**Results:**

HOMA-IR was significantly correlated with hepatic triglyceride content (r:0.76; p<0.0001). The lean-with-NAFLD group had significantly higher HOMA-IR (p<0.001) and lower serum adiponectin (p<0.05) than the overweight-without-NAFLD group. Insulin resistance was independently associated with NAFLD but not with waist circumference or BMI. Regression analysis showed hepatic triglyceride content to be the most important determinant of insulin resistance (p<0.01).

**Conclusions:**

Our findings suggest that NAFLD, once established, seems to be involved in insulin resistance and cardio-metabolic risk factors above and beyond waist circumference and BMI in non-obese, non-diabetic Caucasian individuals.

## Introduction

Insulin resistance is the pathophysiological precursor of type 2 diabetes mellitus (DM-2). Its relationship with non-alcoholic fatty liver disease (NAFLD) has been widely studied in patients with obesity, metabolic syndrome (MS) [1], and DM-2 [2], reaching a prevalence of 60–90% in these populations [3], suggesting that increased fat accumulation in the liver may be an independent risk factor for the development of insulin resistance, DM-2 [2] and cardiovascular disease (CVD) [4,5]. It is well documented that patients with NAFLD more frequently die from CVD than from chronic liver disease [6,7]. NAFLD has been extensively studied using not only ultrasound but also liver biopsies or proton magnetic resonance spectroscopy (H^1^-MRS) in obesity and diabetes [1]. H^1^-MRS is the noninvasive gold standard for the quantification of liver triglyceride content [8,9] and has been widely validated in population-based studies [8] and clinical trials [10]. In contrast, ultrasound imaging is an operator-dependent method with high intra-observer and inter-observer variability [11] that offers inadequate sensitivity and specificity in cases of mild or moderate steatosis [12,13]. However, insulin resistance and NAFLD are also found in non-obese individuals [14,15], and most research in non-obese subjects has been carried out in Asia in lean individuals using ultrasound [15–17] and few studies have employed H^1^-MRS [18,19]. In addition, we have found no publications on insulin resistance and NAFLD in overweight Western individuals, (body mass index [BMI] of 25–30 kg/m^2^) as a single group, and there are no studies in lean Caucasian individuals using H^1^-MRS or liver biopsies for the quantification of hepatic triglyceride content. In fact, there have only been two studies in lean Western subjects (BMI 18.5–24.9 kg/m^2^), and these used only serum aminotransferase levels and/or ultrasound to assess liver fat content [14,20]. An appreciable number of false-negative results are expected with these methods, given the large proportion of individuals with NAFLD who have “normal” serum aminotransferase levels, alanine aminotransferase (ALT) <40 IU [1,21,22] and the poor sensitivity of abdominal ultrasound to detect mild steatosis (liver fat content<30%) [12,13]. This is of special importance in studies of non-obese populations, which are likely to contain a larger proportion of individuals with mild steatosis in comparison to obese subjects. Waist circumference is a simple, indirect method for measuring visceral adiposity. Although it may represent visceral and subcutaneous fat, it has been described as the most reliable surrogate marker of visceral adiposity [23], and a strong correlation has been reported between waist circumference and visceral adiposity assessed with Magnetic Resonance Imaging [24]. It has long been debated whether insulin resistance precedes or is a consequence of NAFLD. It is now generally accepted that excess energy intake initially induces the accumulation of fat (mainly subcutaneous) and insulin resistance, with a subsequent increase in intrahepatic triglyceride content [25]. We also included BMI in this study because it has been related by observational and epidemiological studies to morbidity and mortality risk, highlighting the finding of an increased risk of cardiovascular disease with higher BMI in all population groups. In addition, BMI has been shown to be the best single predictor of total adipose tissue volume assessed with computed tomography in male and female adults [26].

In short, inadequate data are available on insulin resistance in lean and overweight Caucasian individuals and its possible relationship with liver fat content, waist circumference (as proxy of visceral adiposity), BMI, and cardiometabolic risk factors.

With this background, our aim was to study insulin resistance in non-obese, non-diabetic Caucasian individuals and investigate its relationship with liver triglyceride content, waist circumference (proxy of visceral adiposity), and BMI, avoiding the potential confounding factors of diabetes and obesity and using 3T H^1^-MRS to accurately quantify liver triglyceride content.

## Materials and methods

### Study population

A total of 113 healthy, non-obese, non-diabetic, Caucasian adults, aged between 25 and 70 years, participated in this study. Participants were consecutively recruited among individuals undergoing examination at the Occupational Risk Prevention Unit in Granada (Southern Spain) for routine annual general checkup.

Study exclusion criteria were: history of daily alcohol intake > 20 g (men) or > 10 g (women), based on responses to a validated questionnaire on alcohol consumption and confirmation of results by a family member; the presence of HBV/HCV serologic markers, autoimmune hepatitis, primary biliary cirrhosis, hemochromatosis, Wilson’s disease, cancer, diabetes mellitus, or endocrinal, cardiac, renal, or pulmonary disease; consumption of drugs that might cause steatosis (e.g. corticosteroids, amiodarone, methotrexate, tamoxifen); BMI <18.5 or ≥30 kg/m^2^; and the wearing of a pacemaker or other device incompatible with ^1^H-MRS. The study was approved by the ethics committee of San Cecilio University Hospital.

### Study design, anthropometric evaluations and groups

Participants attended two appointments within < 7 days. The first visit involved a full medical history, physical examination, blood analyses and abdominal ultrasound as part of the screening process. Waist circumference was measured with soft tape midway between the lowest rib and the iliac crest in standing position. The weight and height of participants were recorded, calculating their BMI (kg/m^2^). Following WHO criteria for Western populations, a lean individual was defined by a BMI of 18.5–24.9 kg/m^2^ and an overweight individual by a BMI of 25–29.9 kg/m^2^. At the second visit, blood was drawn in the morning after overnight fasting, and their hepatic triglyceride content was quantified by 3 Tesla Magnetic Resonance Spectroscopy (3T H^1^-MRS).

Four groups were then established to study the relationship of insulin resistance with hepatic triglyceride content and cardio-metabolic risk factors in overweight and lean individuals, based on their BMI and the presence or absence of NAFLD (defined by hepatic triglyceride content >5.56% as quantified by 3T H^1^-MRS): *Lean-with-NAFLD*, *Lean-without-NAFLD*, *Overweight-with-NAFLD*, and *Overweight-without-NAFLD*. Based on a previous study with similar methodology and non-obese participants (19), and on a pilot sample of 20 cases, a total sample size of 96 cases was estimated, assuming an α-error of 0.05 and power (1-β) of 0.8. The sample size for each group was therefore set at 25–30 individuals. Once the 3T H^1^-MRS result was obtained, participants were consecutively allocated to the corresponding group until the sample size was reached for each group. All participants received complete information and gave written informed consent to participate in the study.

### Laboratory analysis

Serum ALT and aspartate aminotransferase (AST) levels were determined by a kinetic method (Cobas c 311, Roche Diagnostics GmbH, Mannheim, Germany), with coefficients of variation of 3.3 and 3.1, respectively, serum glucose by the glucose oxidase (enzymatic) method (Roche/Hitachi Analytics systems, Roche Diagnostics GmbH), adiponectin levels by radioimmunoassay, (Linco Research, St. Charles, MO, USA), serum Insulin by electrochemiluminescence immunoassay (Elecsys 2010, Roche Diagnostics GmbH), serum TNF-α by human TNF-alpha enzyme-linked immunosorbent assay (Biosource Europe, Nivelles, Belgium), and serum cholesterol by an enzymatic method (Roche Diagnostics GmbH). Insulin resistance was calculated as HOMA-IR = fasting insulin (mU/L) x fasting glucose (mmol/L)/22.5 [27]. Coefficients of variation in the biochemical tests ranged from 3.1 to 9.9%.

### 3 Tesla H^1^-MRS analysis

A magnetic resonance imaging study was conducted before the spectroscopy, acquiring in-vivo spectra at 3T with a Philips Achieva system (Royal Philips, Amsterdam, Netherlands). A 3- plane localizer was employed to plan the 1H-MRS, and the spectra were obtained using the body coil of the scanner. Breath-hold was monitored using a respiratory belt.

A single voxel of 27cm3 (30 x 30 x 30mm) was selected within normal liver tissue in segment VI, avoiding the edge of the liver, the diaphragm, and major blood vessels. All spectra were obtained with a stimulated echo acquisition mode sequence (STEAM), setting the following parameters: repetition time = 8000; echo time = 20, 40, and 60ms; number of signal averages = 4 (without water suppression); and bandwidth = 2000. Data were acquired within a breath hold. T2 correction was applied and field homogeneity was adjusted automatically for each voxel.

MRS images were reconstructed with Extended MR WorkSpace software (Royal Philips). Raw data were zero-filled once, with no filter, and were phase-corrected, Fourier-transformed, baseline-corrected, and averaged. A Marquardt curve was fitted, using a combined Lorentzian–Gaussian model to calculate the area under the curve of fat and water peaks. Spectra were referenced to residual water and the dominant methylene lipid (–CH2) peak at δ = 4.47 and δ = 1.43 ppm, respectively. Fat fraction percentage (FF) was defined as FA / (FA+WA) x 100, where FA is the area under the fat peak and WA is the area under the water peak. ^1^H-MRS data were interpreted by an experienced radiologist blinded to the biochemical results.

NAFLD was defined by an hepatic triglyceride content greater than 5.56%, which corresponds to 5.56 g/100g (g triglyceride per 100 g wet liver tissue), as previously proposed [8].

### Statistical analysis

Results were expressed as means ± standard deviation (SD). The Kolmogorov-Smirnoff test was used to check the normality of the data distribution. Mean values were compared among groups with the one-way ANOVA, followed by the Tukey multiple-comparison test, the unpaired Student’s two-tailed t test or nonparametric Mann-Whitney U test, as appropriate. Correlations were examined by Pearson standard linear regression analysis (normal distribution) or by the Spearman test (non-normal distribution). The chi-square test was used for non-continuous variables.

Regression analyses were conducted on the global population to increase the sample size and therefore statistical power for evaluation of the main predictors of insulin resistance and NAFLD in our population of non-obese, non-diabetic individuals. Backward stepwise multiple regression analysis was performed to establish the most significant determinants of insulin resistance, entering the following variables: age, sex, hepatic triglyceride content, waist circumference, BMI, and serum ALT, AST, GGT, triglyceride, adiponectin, and HDL-cholesterol (HDL-C) values. Backward Wald binary logistic regression analysis was used to study the main predictors of NAFLD, entering the following variables: age, sex, waist circumference, BMI, HOMA-IR, and serum ALT, AST, GGT, fasting insulin, triglyceride, adiponectin, and HDL-cholesterol (HDL-C) values. Only variables with P < 0.05 were retained in the final regression model. Data analyses were performed with SPSS software for Windows version 22 (IBM SPSS Inc., Chicago IL).

## Results

### Anthropometric, biochemical, and metabolic data

The final study sample comprised 113 adults with a mean ± SD age of 45.1± 10.2 years (range, 25–70 yrs). Their anthropometric and biochemical data are exhibited in Table 1.

**Table 1:** Anthropometrical and biochemical parameters in lean and overweight Caucasian individuals.

	Lean n = 55	Overweight n = 58	P<
Sex (m/f)	28/27	31/27	NS
Age (years)	41.35±10.29	46.25±11.08	0.05
Body Mass Index (Kg/m^2^)	23.52±1.75	27.53±1.44	0.001
Waist Circumference (cm)	85.03±8.12	97.34±10.20	0.001
Hepatic Triglycerides (%)	13.02±6.65	18.29±8.43	0.01
Cholesterol (mg/dl)	191.42±37.15	194.28±44.16	NS
LDL (mg/dl)	108.85±29.91	115.59±37.98	NS
HDL (mg/dl)	66.59±14.28	53.77±13.78	0.05
Triglycerides (mg/dl)	76.12±39.34	117.35±77.64	0.05
Serum AST (IU/L)	31.21±14.89	23.96±6.31	NS
Serum ALT (IU/L)	30.62±23.22	30.77±10.11	NS
Serum GGT (IU/L)	37.05±58.75	70.22±32.89	0.05
Glucose (mg/dl)	92.05±11.27	102.40±19.05	NS
Fasting serum insulin (μU/ml)	8.16±5.01	9.80±4.37	NS
HOMA-IR	3.51±2.33	4.93±2.96	NS
TNF-α (ρg/ml)	148.97±41.27	160.34±40.38	NS
Adiponectin (μg/ml)	13.25±8.16	10.98±5.82	0.05

ALT, alanine aminotransferase, AST, aspartate aminotransferase; BMI, body mass index; GGT, gamma-glutamyl transferase; HDL-C, high-density lipoprotein cholesterol; HOMA-IR, homeostasis model assessment of insulin resistance; LDL-C, low-density lipoprotein cholesterol; NAFLD, non-alcoholic fatty liver disease; NS, non-significant; TNFα, tumor necrosis factor-α. Data are expressed as means ± standard deviation.

P value: the chi-square test for non-continuous variables and the unpaired Student’s two-tailed t-test or nonparametric Mann-Whitney U test, as appropriate, for continuous variables.

According to the Kolmogorov-Smirnoff test, the only variables that significantly deviated from a normal distribution were intrahepatic triglyceride content (p<0.001) and HOMA-IR (p<0.001). Among overweight individuals and lean individuals, considered separately, significant differences were found in BMI, waist circumference, and hepatic triglyceride content (Table 1), using the unpaired Student’s two-tailed t test or nonparametric Mann-Whitney U test, as appropriate.

As observed in Table 2, the mean age, BMI, waist circumference, serum ALT, AST, GGT, triglyceride levels, fasting serum insulin, and HOMA–IR were significantly higher and adiponectin and HDL-C values were significantly lower in subjects with than without NAFLD, considered globally, using the unpaired Student’s two-tailed t-test or non-parametric Mann-Whitney U test, as appropriate.

**Table 2:** Anthropometrical and biochemical parameters of non-obese Caucasian individuals according to the presence of NAFLD.

	No NAFLD n = 58	NAFLD n = 55	P<
Sex (m/f)	27/31	32/23	0.05
Age (years)	42.05±10.16	47.59±10.24	0.01
Body Mass Index (Kg/m^2^)	24.87±2.71	27.28±2.27	0.001
Waist Circumference (cm)	87.65±11.68	98.91±9.88	0.001
Hepatic Triglycerides (%)	2.37±1.39	28.94±13.82	0.001
Cholesterol (mg/dl)	192.26±43.30	194.54±40.39	NS
LDL-C (mg/dl)	112.86±35.07	114.43±36.03	NS
HDL-C (mg/dl)	61.23±14.91	51.73±15.14	0.01
Triglycerides (mg/dl)	85.81±65.75	130.50±70.09	0.01
Serum AST (IU/L)	21.86±8.02	29.05±11.05	0.01
Serum ALT (IU/L)	20.05±6.97	41.30±18.14	0.001
GGT (IU/L)	27.91±24.23	56.19±53.74	0.01
Glucose (mg/dl)	94.86±15.44	105.89±50.28	NS
Fasting serum insulin (μU/ml)	5.92±2.20	12.91±6.90	0.001
HOMA-IR	2.47±1.04	6.73±4.10	0.001
TNF-α (ρg/ml)	150.62±27.41	163.32±53.04	NS
Adiponectin (μg/ml)	14.81±8.60	7.57±7.06	0.001

ALT, alanine aminotransferase, AST, aspartate aminotransferase; BMI, body mass index; GGT, gamma-glutamyl transferase; HDL-C, high-density lipoprotein cholesterol; HOMA-IR, homeostasis model assessment of insulin resistance; LDL-C, low-density lipoprotein cholesterol; NAFLD, non-alcoholic fatty liver disease; NS, non-significant; TNFα, tumor necrosis factor-α. Data are expressed as means ± standard deviation.

P value: the chi-square test for non-continuous variables and the unpaired Student’s two-tailed t-test or nonparametric Mann-Whitney U test, as appropriate, for continuous variables.

In comparison to the lean-NAFLD group, significantly higher HOMA-IR, hepatic triglyceride content, waist circumference, BMI, and serum triglyceride values and significantly lower serum adiponectin and HDL-C levels were observed in the overweight-with-NAFLD group (Table 3)

**Table 3:** Anthropometrical and biochemical parameters in lean and overweight Caucasian individuals according to the presence of NAFLD.

	LeanwithoutNAFLDn = 30	LeanwithNAFLDn = 25	OverweightwithoutNAFLDn = 28	OverweightwithNAFLDn = 30
Age (years)	39.71±7.30	43.00±12.81	44.27±12.04	48.24±9.61^a^
Sex (m/f)	14/16	14/11^a^	13/15	18/12^a^^.^^c^
Body Mass Index (Kg/m^2^)	22.59±1.61	23.46±1.96	27.06±1.41^d^^.^^e^	28.00±1.46^c^^.^^d^^.^^e^
Waist Circumference (cm)	81.19±8.74	88.86±7.95^a^	93.82±10.9^d^	100.86±9.07^c^^.^^d^^.^^e^
Hepatic Triglycerides (%)	2.15±1.34	23.90±11.91^d^^.^^f^	2.60±1.43	33.98±15.24^d^^.^^e^^.^^f^
Cholesterol (mg/dl)	189.24±41.02	193.60±32.22	195.14±46.15	193.43±41.87
LDL-C (mg/dl)	108.71±31.61	109.00±28.22	116.82±38.41	114.36±37.09
HDL-C (mg/dl)	68.80±13.33	64.38±15.59	58.23±13.93	49.31±13.69^c^^.^^d^^.^^e^
Triglycerides (mg/dl)	72.05±37.86	80.20±41.57	98.95±83.14	135.75±70.70 ^b^^.^^d^
Serum AST (IU/L)	23.10±10.46	39.33±17.60^d^	20.68±4.62^e^	27.24±8.71^c^^.^^a^
Serum ALT (IU/L)	19.38±7.93	41.86±38.59^d^	20.68±6.02^e^	40.87±17.44^f^^.^^d^
Serum GGT (IU/L)	26.29±24.02	47.81±40.16^d^	29.45±24.90^e^	111.00±89.54^c^^.^^a^
Fasting Glucose (mg/dl)	90.71±6.12	93.40±16.23	98.82±18.19	105.99±20.29^a^
Fasting Insulin (μU/ml)	5.62±2.16	10.70±7.60^a^	6.20±2.27^b^	13.41±6.96^d^^.^^f^
HOMA-IR	2.26±0.96	4.75±3.46^d^^.^^f^	2.68±1.10	7.18±4.22^b^^.^^d^^.^^f^
TNF-α (ρg/ml)	146.54±24.41	151.40±67.27	154.51±30.04	166.17±50.17^a^
Adiponectin (μg/ml)	14.24±9.05	12.27±8.08^a^^.^^c^	15.35±7.15	6.61±4.58^d^^.^^e^^.^^f^

ALT, alanine aminotransferase, AST, aspartate aminotransferase; BMI, body mass index; GGT, gamma-glutamyl transferase; HDL-C, high-density lipoprotein cholesterol; HOMA-IR, homeostasis model assessment of insulin resistance; LDL-C, low-density lipoprotein cholesterol; NAFLD, non-alcoholic fatty liver disease; TNFα, tumor necrosis factor-α.

Data are expressed as means with standard deviation (SD).

^a^ P< 0.05 vs. Lean-without-NAFLD.

^b^ P< 0.05 vs. Lean-with-NAFLD.

^c^ P< 0.05 vs. Overweight-without-NAFLD.

^d^ P< 0.001 vs. Lean- without-NAFLD.

^e^ P< 0.001 vs. Lean-with-NAFLD.

^f^ P< 0.001 vs. Overweight-without-NAFLD.

^a-f^ P value: chi-square test for non-continuous variables and one-way ANOVA for continuous variables followed by the Tukey multiple-comparison test

**Fig 1:**
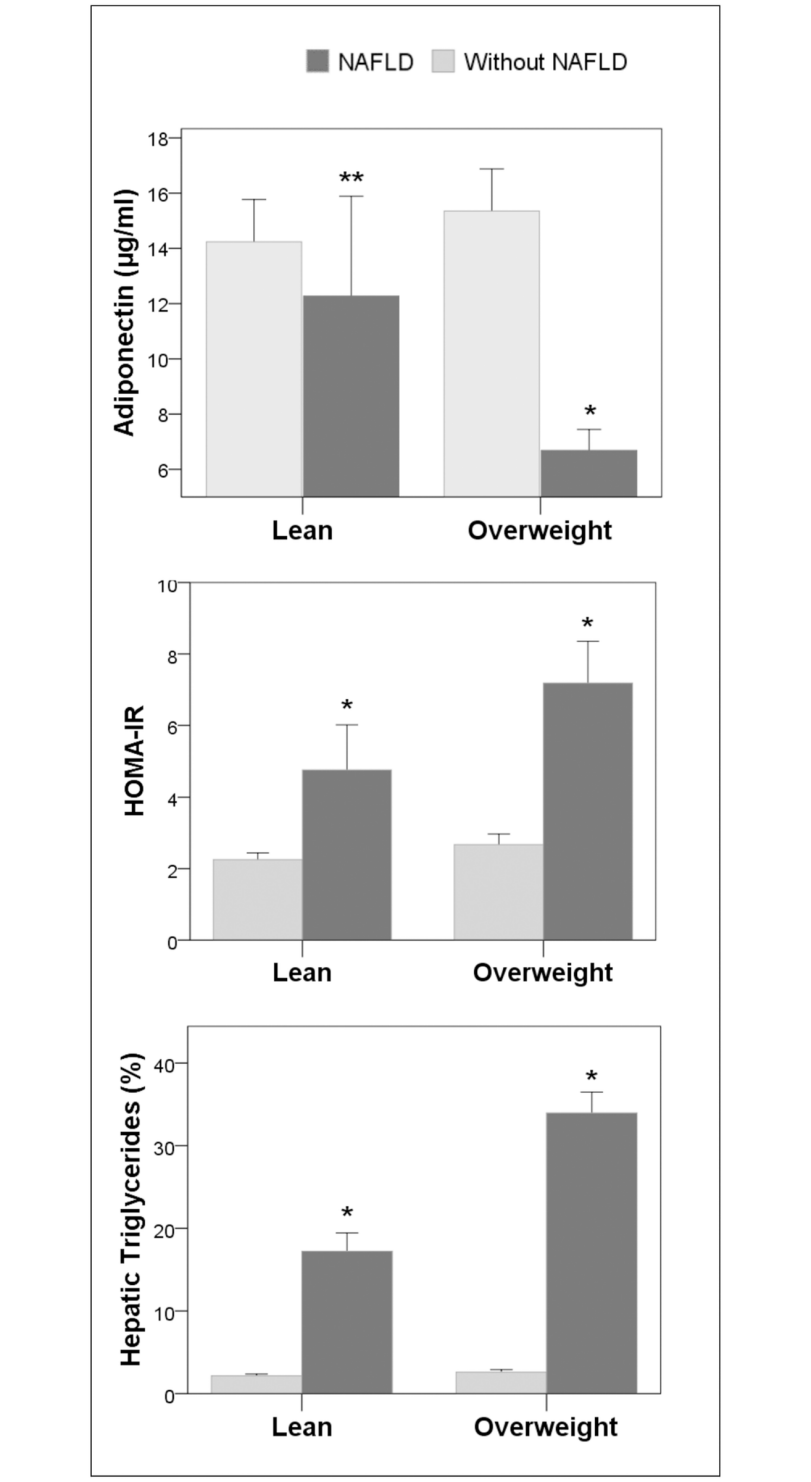
Comparison of HOMA-IR, serum adiponectin level and hepatic triglyceride content between lean and overweight groups with and without NAFLD. Changes in HOMA-IR and serum adiponectin level paralleled changes in hepatic triglyceride content. *: p< 0.001 *versus* all other groups. **: p< 0.05 *versus* lean- and overweight-without NAFLD groups.

The only significant differences found between the overweight-without-NAFLD group and the lean-without-NAFLD group were the higher waist circumference and BMI in the former. Finally, in comparison to the overweight-without-NAFLD group, the lean with NAFLD group had significantly higher hepatic triglyceride content, HOMA-IR, and serum fasting insulin, AST and ALT values and significantly lower serum adiponectin values (using one-way ANOVA for continuous variables followed by the Tukey multiple-comparison test). As depicted in Fig 1, changes in HOMA-IR and serum adiponectin levels paralleled changes in hepatic triglyceride content in all groups.

### Correlations of HOMA-IR

HOMA-IR was highly significantly and positively correlated with hepatic triglyceride content (r:0.76;p<0.0001), and other less highly significant positive correlations were found with waist circumference (r:0.52;p<0.001), BMI (r:0.48;p<0.01), age (r:0.49;p<0.01) and serum ALT (r:0.3;p<0.03), while inverse correlation with serum adiponectin (r:0.47;p<0,01) and HDL-C (r:0.46;p<0.02) were observed.

### Correlations of hepatic triglyceride content with metabolic risk factors and other variables

Hepatic triglyceride content was highly significantly and positively correlated with HOMA-IR, (r:0.76;p<0,0001), fasting serum insulin (r:0.71, p<0.0001) and serum ALT (r:0.63; p< 0.001). Other less highly significant positive correlations were found with waist circumference (r:0,48;p<0.01), BMI (r:0.39, p<0.01), and serum triglycerides (r.0.41, p<0.01), while inverse correlations with serum adiponectin (r:0.01, p<0.46) and HDL-C (r:0.39; p<0.01) were observed.

### Backward stepwise multiple regression analysis of factors associated with insulin resistance

In the regression analyses on insulin resistance predictors, hepatic triglyceride content was the most influential determinant (*B*-coefficient = 0.16, SE = 0.017; p<0.01), while the effect of age was also significant (*B*-coefficient = 0.06, SE = 0.019; p<0.05). Only significant variables (p<0.05) are shown.

### Backward wald binary logistic regression analysis on factors associated with NAFLD

Backward stepwise regression analyses showed that the most influential determinants of NAFLD in our sample of non-obese individuals were HOMA-IR (*B*-coefficient = 2.9, SE = 1.5; (p<0.01) and serum ALT (*B*-coefficient = 0.55, SE = 0.25; p<0.032). Only significant variables (p<0.05) are shown.

## Discussion

The results of this study indicate that increased hepatic triglyceride content is closely associated with insulin resistance in non-obese, non-diabetic Caucasian individuals above and beyond waist circumference (proxy of visceral adiposity) and BMI. Overfeeding studies have shown that subcutaneous fat accumulation and insulin resistance are early effects of positive energy balance, and that hepatic triglyceride accumulation occurs later [28,29]. However, the relative importance of the relationships of NAFLD, waist circumference and BMI with insulin resistance and other cardiovascular risk factors remains to be elucidated [1,30–32].

To our best knowledge, this is the first published report designed to improve understanding of the clinical significance of NAFLD, waist circumference, and BMI in relation to insulin resistance and cardiometabolic risk factors in lean and overweight Caucasian individuals, and using 3T H^1^-MRS for liver fat assessment. Higher insulin resistance values were found in the lean-with-NAFLD group than in the overweight-without-NAFLD group despite the significantly higher waist circumference and BMI of the latter. This suggests that the increased hepatic triglyceride accumulation in the lean-with-NAFLD group rather than their waist circumference or BMI contributes to the development of insulin resistance. In addition, no differences in insulin resistance or the other metabolic risk factors were found between the lean and overweight individuals without NAFLD, who only differed in waist circumference and BMI. This supports the proposal that insulin resistance is more closely associated with NAFLD than with waist circumference or BMI. Nevertheless, our correlation analyses indicate that an association of both BMI and waist circumference with insulin resistance and other metabolic risk factors can be expected. Similar results were reported for females but not males in a previous study; this discrepancy with our findings may be explained by their use of computed tomography and their inclusion of obese subjects [32]. In addition, multiple regression analysis showed that hepatic triglyceride content was the most important determinant of insulin resistance, even after adjustment for sex and age, while neither waist circumference nor BMI were significant predictors, which supports the concept of metabolically obese but normal weight individuals, with normal BMI but significant risk factors for diabetes, metabolic syndrome, and cardiovascular disease [33]. Based on these findings, we suggest that NAFLD, once established, appears to be involved in insulin resistance and cardio-metabolic risk factors above and beyond waist circumference or BMI in non-obese non-diabetic individuals.

Insulin resistance, low serum levels of adiponectin and HDL-C and high serum triglyceride levels have been considered to represent a possible link between NAFLD and atherosclerotic vascular disease [34]. This proposal might be applicable to lean individuals, as supported by the present study in non-obese subjects, in which adiponectin, lipid profile abnormalities and insulin resistance were significantly associated with the increased accumulation of hepatic triglycerides.

Elevated serum TNF levels have been observed in chronic liver disease [35,36] and in some cases of NAFLD [20,37]. In our study, TNFα serum levels were higher in the overweight-with-NAFLD group than in the lean-without-NAFLD group but showed no correlation with hepatic triglyceride content, insulin resistance, or serum aminotransferase levels. The implication of the TNF system in the development of insulin resistance and other metabolic consequences in non-obese, non-diabetic individuals remains to be elucidated.

Study limitations include a potential selection bias, in that individuals undergoing a routine general checkup may be more health conscious than the general population. In addition, it was not possible to investigate the natural progression of insulin resistance due to the cross-section design of the study. The sample size delivered adequate statistical power, but further studies in wider samples are warranted to verify these findings.

Study strengths include the use of 3T H^1^-MRS for liver triglyceride quantification, the prospective enrolment of patients, the strict exclusion criteria imposed, and the gathering of all biochemical and 3T H^1^-MRS measurements within a 24-h period.

In conclusion, our findings suggest that NAFLD, once established, appears to make a greater contribution to insulin resistance and cardio-metabolic risk factors in comparison to waist circumference and BMI in non-obese, non-diabetic Caucasian individuals.

## Supporting information

S1 File Dataset providing additional relevant data. (XLSX)
